# Childhood vaccinations: knowledge, attitudes and practices of paediatricians and factors associated with their confidence in addressing parental concerns, Italy, 2016

**DOI:** 10.2807/1560-7917.ES.2019.24.6.1800275

**Published:** 2019-02-07

**Authors:** Antonietta Filia, Antonino Bella, Fortunato D’Ancona, Massimo Fabiani, Cristina Giambi, Caterina Rizzo, Lorenza Ferrara, Maria Grazia Pascucci, Maria Cristina Rota

**Affiliations:** 1 Department of Infectious Diseases, Istituto Superiore di Sanità, Rome, Italy; 2 Regional Epidemiology Unit for Infectious Diseases (SeREMI), ASL, Alessandria, Italy; 3 Regional Health Authority, Emilia-Romagna Region, Bologna, Italy

**Keywords:** vaccinations, paediatricians, knowledge, attitudes, practices, hesitancy

## Abstract

**Background:**

Paediatrician recommendations are known to influence parental vaccine decisions.

**Aim:**

Our aim was to examine vaccination knowledge, attitudes and practices among paediatricians in Italy and identify factors associated with their confidence in addressing parental questions.

**Methods:**

An electronic questionnaire survey was conducted from February to March 2016, among a sample of Italian paediatricians.

**Results:**

The survey was completed by 903 paediatricians (mean age: 56 years). Of 885 who responded to the specific question, 843 (95.3%) were completely favourable to vaccinations. Sixty-six per cent (570/862) felt sufficiently knowledgeable about vaccinations and vaccine-preventable diseases to confidently discuss them with parents. Paediatricians who were male, who were 55 years or older, who had participated in training courses in the last 5 years, who reported that taking courses and reading the scientific literature had contributed to their knowledge, or who had implemented vaccination promotion activities, felt more knowledgeable than other paediatricians. When asked to rate their level of agreement with statements about vaccine safety and effectiveness, only 8.9% (80/903) responded fully as expected. One third (294/878) did not systematically verify that their patients are up to date with the immunisation schedule. Only 5.4% (48/892) correctly identified all true and false contraindications.

**Conclusions:**

The majority of paediatricians in Italy are favourable to vaccination but gaps were identified between their overall positive attitudes and their knowledge, beliefs and practices. Targeted interventions are needed aimed at increasing paediatricians’ confidence in addressing parents’ concerns, strengthening trust towards health authorities and improving systems barriers.

## Background

Vaccination is one of the most important public health measures developed in the history of medicine, allowing for the primary prevention of serious infectious diseases. Currently, many countries in Europe and worldwide, including Italy, are facing declining childhood vaccination rates. This poses a threat to herd immunity and increases the risk for outbreaks of vaccine-preventable diseases (VPD). Vaccine hesitancy, defined by the Strategic Advisory Group of Experts on Immunization (SAGE) as a “delay in acceptance or refusal of vaccines despite availability of vaccination services” is believed to be one of the reasons for the decreasing coverage [1]. According to SAGE, “vaccine hesitancy is complex and context specific varying across time, place and vaccines. It is influenced by factors such as complacency, convenience and confidence” [1]. Vaccine-hesitant individuals may accept some vaccines and refuse or delay others, although some remain unsure about their decision. Some studies have identified vaccine safety concerns as the main reason for not vaccinating or delaying vaccinations [2-4].

Healthcare workers (HCW) are considered the most trusted source of information on vaccines by parents, and their recommendations are known to influence parental vaccine decisions [5,6]. Paediatricians in particular are in a good position to explain to parents the risks of VPD and the benefits and risks of vaccination, and to understand and respond to worries and concerns that parents may have about vaccinating their children. However, HCW, including paediatricians, may themselves have concerns regarding the usefulness of vaccines and vaccine side effects and be vaccine-hesitant regarding vaccinations for themselves, their children or their patients [5-7]. Their beliefs and attitudes may lead them to recommend vaccines to their patients less frequently and may have a negative influence on parents’ vaccination acceptance [8]. Few published studies from the European Union (EU) have evaluated paediatricians’ knowledge, attitudes and practice regarding VPD and vaccines [9,10].

In Italy, everyone must be registered with a primary care provider in order to access healthcare services of the national healthcare system. Primary care paediatricians (PCP) provide general and preventive (well-child) healthcare to children up to 14 years of age but most usually do not provide vaccinations directly to the children registered in their practices. Childhood vaccinations are publicly funded, provided free of charge mainly at vaccination centres managed by local health authorities, and administered mainly by public health physicians or nurses. In some areas, PCP may support vaccination services according to local agreements. Community paediatricians work in outpatient services provided by local health authorities and perform various activities, often in collaboration with PCP and other health professionals, including immunisations, health education, parental counselling as well as management of chronic illnesses and of behavioural and developmental problems. Paediatricians in private practice may deliver vaccinations in their offices.

Overall vaccination rates for most VPD (tetanus, diphtheria, pertussis, poliomyelitis, hepatitis B and *Haemophilus influenza*e type b), but not for measles, have historically been very high in Italy and this has resulted in either elimination or a decreased incidence of these diseases. However, since 2013, uptake has been steadily decreasing and pockets of vaccination opponents exist in some Regions [2]. In 2017, more than 5,000 measles cases including four deaths were reported in Italy, 93% of which occurred in persons that were either not vaccinated or vaccinated with only one dose of measles-containing vaccine. About 25% of measles cases occurred in children up to 14 years of age [11,12].

Given the critical role that paediatricians play in providing information to parents about childhood vaccinations, we performed a study to examine vaccination knowledge, attitudes and practices among paediatricians in Italy, and to identify factors associated with their feeling sufficiently knowledgeable about vaccinations and VPD to be able to confidently address parental questions. The survey was part of a wider project funded by the Ministry of Health, aiming to describe vaccine refusal in Italy and to prepare ad hoc communication tools. It was conducted before the introduction in Italy of a law extending the number of mandatory vaccinations from four (poliomyelitis, tetanus, diphtheria and hepatitis B) to 10 (pertussis, *Haemophilus influenza* type b vaccine, measles-mumps-rubella and varicella, in addition to the four already mandatory) in children up to 16 years of age. Since September 2017, proof of vaccination has been required to attend kindergarten and nurseries; lack of compliance in older children does not limit their access to school, but financial sanctions are applied to parents refusing vaccination.

## Methods

A self-completed electronic survey, developed with the SurveyMonkey internet platform (San Mateo, California, United States (US)), was conducted from February to March 2016 among Italian paediatric specialists registered with any of the five main scientific paediatric societies in Italy. About 10,000 of the ca 13,000 paediatricians in Italy are members of the largest of the five paediatric societies and ca 90% of the ca 7,700 PCP are members of the second largest society. A third society has 1,400 members, and we are not aware of the total number of members of the two remaining societies. Paediatricians may be members of more than one scientific society.

A two-step procedure was used to enrol paediatricians in the study. Firstly, we contacted the five paediatric societies and asked them to send an email to all their registered members to explain the aims of the survey and ask them whether they were willing to participate. We then sent a link to the electronic questionnaire to those paediatricians who expressed an interest in participating. The survey was completely anonymous and did not collect personal identifiers nor sensitive data; it therefore did not require approval by an Ethics Committee.

### Questionnaire

We developed the questionnaire after reviewing the literature and also used or adapted some questions used in previous studies on this topic [13-15]. The questionnaire consisted of two sections (Supplement). The first section contained 20, mostly closed-ended questions, to collect information about paediatricians’ (i) knowledge regarding vaccine effectiveness and true and false contraindications (questions 15–16), (ii) perceptions regarding frequency and severity of VPD, vaccine safety, parents’ level of concern about vaccination and their reasons for refusing vaccinations for their child (questions 7–8, 13–14, 17), (iii) beliefs and attitudes towards vaccinations (questions 18–19) and (iv) professional experience and practice regarding vaccinations (questions 1–6, 9–12, 20). To assess paediatricians’ beliefs and attitudes towards vaccinations, they were asked to rate their level of agreement with each of 19 statements according to a five-point Likert scale (completely disagree, partially disagree, no opinion, partially agree, completely agree). To evaluate their knowledge regarding vaccine contraindications, they were asked to classify 11 clinical conditions or situations as false contraindications, temporary contraindications or permanent contraindications to administering hexavalent vaccine (containing diphtheria, tetanus, acellular pertussis, poliomyelitis, *Haemophilus influenzae* type b and hepatitis B components).

In the second section, made up of 16 questions (questions 21–36), paediatricians were asked to provide demographic information, information regarding their training, and type and years of practice. We also asked participants whether they considered themselves sufficiently knowledgeable about vaccines and VPD (including incidence, complications, contraindications and vaccine benefits and risks) to be able to confidently address parental questions, and if not, for which of eight topics (listed in the questionnaire) they would like to receive further training and in which order of priority (1 lowest priority to 8 highest priority). We then calculated an average score for each topic. The questionnaire was pilot-tested for clarity, length and ease of administration among 15 paediatricians in two Italian Regions (Piemonte and Emilia-Romagna).

### Statistical analysis

We show questionnaire responses as absolute frequencies and percentages (categorical variables) and as means with standard deviation (continuous variables). Paediatricians’ demographic and professional characteristics are described.

The association between the outcome ‘feeling knowledgeable about vaccinations’ and other variables was evaluated using the chi-squared test or Fisher’s exact test when appropriate. All variables describing the demographic and professional characteristics of paediatricians and potentially associated with the outcome (p < 0.20 in bivariate analysis) were considered for possible inclusion into the multivariable model. These included: sex, age, practice location, type of paediatric practice, vaccine courses or conferences attended in the previous 5 years, vaccinology training, country where medical degree was obtained, years since medical degree, years since specialty certification, years of activity as a paediatrician, whether the paediatrician directly administers vaccines, degree of influence of each of various sources of information or training opportunities on their knowledge of VPD and whether the responding paediatrician implemented any vaccination initiatives in the previous year to promote vaccine uptake among their patients. Adjusted odds ratios (OR) and their corresponding 95% confidence intervals (CI) were calculated for each variable.

We then determined the final model using a backward selection process according to the likelihood ratio test for goodness-of-fit. All statistical analysis were performed using STATA software version 11.2 (Stata Corporation, College Station, Texas, US).

## Results

### Characteristics of participating paediatricians

**Figure 1:**
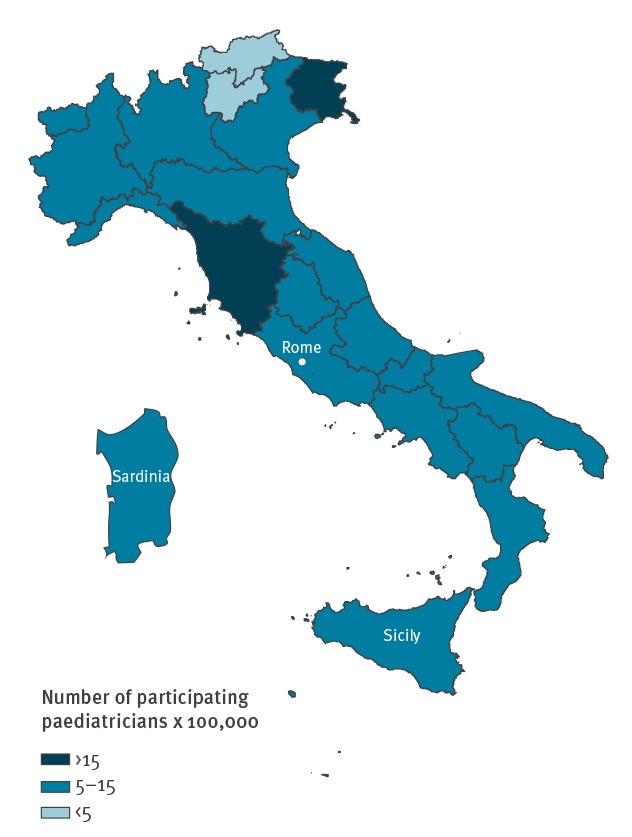
Number of participating paediatricians per 100,000 paediatric population aged 0–14 years, by region, Italy, 2016

The questionnaire was sent to 1,256 paediatricians, of whom 903 (71.9%) returned it with responses and were included in the study. Table 1 summarises their main demographic and professional characteristics: 65.6% (581/885) were 55 years or older (mean age: 56 years), 65.1% (574/882) were women, 78.7% (686/872) had completed postgraduate medical training more than 20 years ago and 71.5% (629/880) were PCP. Practice locations of the responding paediatricians were distributed throughout all 21 Regions of Italy; inside each Region, 91% of the provinces were represented (median: five paediatricians per province; range: 1–73) (Figure 1).

**Table 1:** Main demographic and professional characteristics of participating paediatricians, survey on vaccine knowledge, Italy, 2016 (n = 903)

Characteristic (with number who responded to the specific question)		n	%
Age in years (n = 885)	< 35	39	4.4
	35–44	96	10.8
	45–54	169	19.1
	55–64	487	55.0
	> 64	94	10.6
Sex (n = 882)	Male	308	34.9
	Female	574	65.1
Country where medical degree was obtained (n = 875)	Italy	864	98.7
	Foreign	11	1.3
Years since medical degree (n = 883)	0–5	3	0.3
	6–10	45	5.1
	11–20	94	10.6
	> 20	741	83.9
Years since specialty certification (n = 872)	0–5	63	7.2
	6–10	46	5.3
	11–20	77	8.8
	> 20	686	78.7
Type of practice (n = 880)	Primary care paediatrician	629	71.5
	Community paediatrician	17	1.9
	Hospital paediatrician	161	18.3
	Private practice	57	6.5
	Retired	16	1.8
Practice location (n = 883)	Large city (> 250,000 population)	182	20.6
	Medium-sized city (50,000–250,000 population)	276	31.3
	Small city/town (< 50,000 population)	425	48.1
Years of activity as a paediatrician (n = 882)	< 1	16	1.8
	1–5	61	6.9
	6–10	47	5.3
	> 10	758	85.9
Vaccine courses or conferences in previous 5 years (n = 870)	No	165	19.0
	Yes	705	81.0
Vaccinology training (n = 882)	No	594	67.3
	Yes	288	32.7
Degree of influence of formal university training (n = 717)	Low	252	35.1
	High	465	64.9
Degree of influence of the scientific literature (n = 780)	Low	40	5.1
	High	740	94.9
Degree of influence of conference participation (n = 803)	Low	29	3.6
	High	774	96.4
Degree of influence of institutional websites (n = 652)	Low	133	20.4
	High	519	79.6
Degree of influence of non-institutional websites (n = 596)	Low	470	78.9
	High	126	21.1
Degree of influence of discussions with other colleagues (n = 679)	Low	107	15.8
	High	572	84.2
Implemented vaccination initiatives (n = 901)	No	250	27.7
	Yes	651	72.3
Administers vaccines (n = 896)	No	479	53.5
	Occasionally	302	33.7
	Regularly	115	12.8

Denominators differ for each characteristic as not all paediatricians responded to all questions.

### Training and knowledge

**Figure 2:**
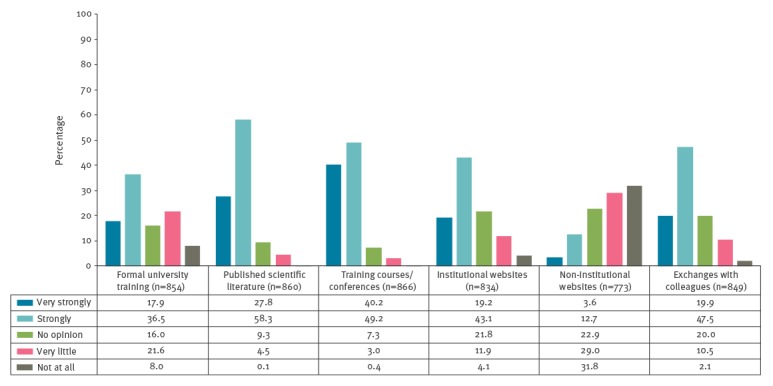
Paediatricians’ responses to the question ‘How much do/have the following six training tools influence/influenced your knowledge on vaccine-preventable diseases?’, Italy, 2016 (n = 903)

Two thirds of paediatricians (570/862) felt sufficiently knowledgeable about vaccinations and VPD to be able to confidently discuss them with parents. When asked to indicate how much each of six possible training tools/settings influenced their knowledge of vaccines and VPD, 30% (252/854) reported that they had received either very little or no formal training in vaccinology during their university studies. Participation in immunisation conferences and courses played an important role for most paediatricians (Figure 2).

Eighty-one per cent of paediatricians (705/870) reported having attended conferences or courses in the previous five years, of whom 42.7% (301/705) completed four or more training courses. The five main topics for which paediatricians who do not feel knowledgeable about vaccinations would like to obtain additional training in were, in order of priority, safety of vaccines (priority score: 5.25), how to respond to parental concerns (4.97), VPD epidemiology and complications (4.84), vaccine contraindications (4.74) and vaccine efficacy (4.70). Only 5.4% of paediatricians (48/892) correctly identified all 11 true and false contraindications to hexavalent vaccine (diphtheria, tetanus, acellular pertussis, poliomyelitis, *Haemophilus influenzae* type b, hepatitis B) listed in Table 2; 44.2% (394/892) correctly classified at least nine of 11 contraindications, 91.5% (816/892) at least six, and 8.5% (76/892) less than six.

**Table 2:** Paediatricians’ responses to the question ‘Your patient is scheduled to receive the second dose of hexavalent vaccine^a^; which of the following conditions do you consider to be a contraindication?’, Italy, 2016 (n = 892)

Condition	False contraindication		Temporary contraindication		Permanent contraindication		Don’t know		Total
	n	%	n	%	n	%	n	%	
Severe allergic reaction to a previous dose, including anaphylaxis	26	3.0	133	15.2	**693**	**79.1**	24	2.7	876
Fever following a previous dose	**831**	**94.6**	38	4.3	7	0.8	2	0.2	878
Acute severe gastroenteritis	431	49.2	**437**	**49.9**	6	0.7	2	0.2	876
Otitis media, without fever	**553**	**63.2**	319	36.5	1	0.1	2	0.2	875
Family history of adverse reaction following a pertussis vaccine dose	**717**	**82.2**	62	7.1	40	4.6	53	6.1	872
Acute upper airway infection, without fever	**629**	**72.0**	236	27.0	8	0.9	1	0.1	874
History of pertussis	**636**	**73.1**	192	22.1	21	2.4	21	2.4	870
Diagnosis of epilepsy, well controlled	**790**	**90.4**	52	5.9	13	1.5	19	2.2	874
Fever 38–40 °C and moderate illness	67	7.7	**796**	**91.0**	10	1.1	2	0.2	875
Fever > 40 °C and severe illness	30	3.4	**811**	**92.0**	37	4.2	4	0.5	882
Congenital immunodeficiency	**251**	**28.7**	64	7.3	460	52.6	99	11.3	874

Correct responses are indicated in bold. Denominators differ for each contraindication as not all paediatricians responded to all contraindications.

^a^ Diphtheria, tetanus, acellular pertussis, poliomyelitis, *Haemophilus influenzae* type b, hepatitis B.

Most paediatricians considered vaccines to be effective or very effective, with a range from 83.5% (738/884) for the human papillomavirus (HPV) vaccine to 99.6% (887/891) for the diphtheria-tetanus-acellular pertussis vaccine.

### Beliefs, attitudes and perceptions

In general, 95.3% (843/885) of paediatricians reported being completely favourable to vaccinations, 3.8% (34/885) were moderately favourable, 0.8% (7/885) had a neutral attitude and one paediatrician (0.1%) was completely against vaccinations. Fifty-eight per cent (512/883) reported being favourable to schools requiring pupils to be vaccinated.

When asked to rate their level of agreement with 19 statements about vaccine safety and effectiveness (Table 3), we would have expected the paediatricians to be in complete disagreement with vaccine-critical statements and in complete agreement with positive statements. However, only 8.9% (80/903) fully responded in that way. For example, a proportion of paediatricians either agreed (completely or partially) or only partially disagreed with the negative statements that “Children receive too many vaccines”, “It is better for children to develop natural immunity rather than to get a vaccine”, “Healthy children do not need to be vaccinated” and “Conditions such as autism and multiple sclerosis may be caused by vaccines” (Table 3). On the other hand, only about two thirds completely agreed with either of the positive statements “Vaccines are among the safest and most tested medicinal products” and “Vaccine information provided by health authorities and scientific societies is reliable”.

**Table 3:** Paediatricians’ attitudes towards vaccination, Italy, 2016 (n = 903)

Questionnaire statements	Completely disagree		Partially disagree		Unsure		Partially agree		Completely agree		Total
	n	%	n	%	n	%	n	%	n	%	
Vaccines weaken or overload the immune system	808	91.5	35	4.0	8	0.9	21	2.4	11	1.2	883
It is better for children to develop natural immunity by getting sick rather than to get a vaccine	707	80.2	102	11.6	11	1.2	49	5.6	13	1.5	882
Healthy children do not need to be vaccinated	835	94.7	22	2.5	2	0.2	7	0.8	16	1.8	882
Conditions such as autism and multiple sclerosis may be caused by vaccines	804	91.0	39	4.4	15	1.7	8	0.9	18	2.0	884
Allergies are on the rise because of vaccinations	743	84.3	47	5.3	36	4.1	30	3.4	25	2.8	881
I am afraid that one of my patients may develop a severe adverse reaction following vaccination	550	62.6	162	18.4	39	4.4	99	11.3	29	3.3	879
Children receive too many vaccines	696	79.1	64	7.3	14	1.6	73	8.3	33	3.8	880
Vaccine policy is influenced by financial profits of pharmaceutical companies	394	44.8	190	21.6	88	10.0	174	19.8	33	3.8	879
Childhood vaccines are given too early	739	84.3	42	4.8	21	2.4	40	4.6	35	4.0	877
The frequency of adverse reactions to vaccines is underestimated	446	51.2	185	21.2	78	9.0	116	13.3	46	5.3	871
In the US, paediatricians are increasingly rejecting patients whose parents refuse vaccinations. I agree with this attitude.	236	26.8	154	17.5	54	6.1	199	22.6	236	26.8	879
Vaccination is cost-effective	128	14.8	46	5.3	73	8.4	122	14.1	496	57.3	865
I am favourable to reintroducing mandatory school immunisation requirements	91	10.3	77	8.7	42	4.8	161	18.2	512	58.0	883
Vaccine information provided by health authorities and scientific societies is reliable	51	5.8	48	5.4	20	2.3	185	20.9	580	65.6	884
Vaccines are among the safest and most tested medicinal products	109	2.5	45	5.1	25	2.9	120	13.7	576	65.8	875
The second dose of MMR is useful	56	6.4	15	1.7	16	1.8	63	7.2	727	82.9	877
When children get vaccinated, the whole community benefits	35	4.0	2	0.2	2	0.2	14	1.6	829	94.0	882
If we stop vaccinating. many diseases that have become rare may re-emerge	2	0.2	11	1.3	4	0.5	22	2.5	838	95.6	877
Vaccines are important for my patients’ health	5	0.6	3	0.3	1	0.1	9	1.0	867	98.0	885

MMR: measles-mumps-rubella vaccine; US: United States.

Denominators differ for each characteristic as not all paediatricians responded to all questions.

Most paediatricians (837/899; 93.1%) perceived that, in the previous 2 years, parents had become increasingly worried about vaccinations. Vaccine safety concerns were perceived by most paediatricians (595/899; 66.2%) as the single most important reason for which parents refuse to vaccinate their child.

### Practice

Ninety-nine per cent of paediatricians (891/900) recommend that parents follow the national immunisation schedule, 0.3% (3/900) recommend only compulsory vaccinations and 0.2% (2/900) recommend parents not to vaccinate against any VPD. Four paediatricians (0.5%) responded that they have a neutral attitude and do not express their opinion regarding vaccinations to parents. 

Overall, 89.9% (808/899) reported frequently discussing with parents about the importance of vaccination, 7.8% (70/899) did so occasionally, 1.9% (17/899) only if parents brought up the topic and 0.4% (4/899) never.

Older paediatricians seemed to be more willing to discuss vaccinations with parents than their younger colleagues: 94.5% (549/581) of those 55 years and older reported using every available opportunity to discuss vaccinations, vs 81.3% (247/304) of younger paediatricians (p < 0.001).

When faced with parents who refuse to vaccinate their child against one or more diseases, 97.8% (861/880) of paediatricians stated that they try to change parents’ minds by providing information about vaccines and risks of diseases. The remaining respondents either do not interfere or support parents’ decisions. Instead, when faced with parents who want to delay vaccinations, only 90.1% (790/877) of paediatricians stated that they try to change their minds; no significant differences were found by age, sex, location or type of practice (data not shown).

Only 66.5% (584/878) of paediatricians verify systematically that their patients are up to date with the national immunisation schedule, 28.6% (251/878) verify frequently, 4.7% (41/878) occasionally and 0.2% (2/878) never. No differences by sex were identified. Paediatricians 55 years and older verify their patients’ vaccination status more frequently than their younger colleagues aged 35–54 years (71.9% vs 56.3%; p < 0.001). A higher proportion of PCP systematically verify that their patients are up to date with immunisations, compared with other paediatricians (72.4% and 50.4% respectively; p < 0.001). Also, a higher proportion of those who have participated in training courses or conferences within the previous five years verify immunisation status of their patients systematically or frequently, compared with those who have not (96.6% vs 88.5%; p < 0.001).

Most paediatricians (651/901; 72.3%) reported having implemented vaccination promotion activities in the previous year, including putting posters and information materials in their waiting rooms (491/901; 54.5%), sending reminders by post or phone (50/901; 5.6%), organising meetings with parents or groups of parents (110/901; 12.2%), sending or giving written information materials to parents (39/901; 4.3%), and recommending websites with reliable vaccine information.

### Factors associated with feeling knowledgeable about vaccinations and vaccine-preventable diseases

The percentage of paediatricians who reported feeling knowledgeable about vaccinations and VPD was significantly greater among paediatricians 55 years and older compared with younger paediatricians (74.7% vs 49.5%; p < 0.001), increasing proportionally with the number of years since specialty certification, from 33.9% (21/62) among those who completed their training a maximum of 5 years previously to 73.0% (436/597) among those who completed their training at least 24 years ago (p < 0.001). No differences were found with respect to practice location (large city vs medium-sized city vs small town, defined in Table 1). Most paediatricians (494/567; 87.1%) who felt knowledgeable about vaccinations had attended training courses in the previous 5 years, compared with only 69.3% (203/293) of those who did not feel knowledgeable (p < 0.001).

Table 4 shows the results of the univariate and multivariable logistic regression model of the factors associated with feeling more or less knowledgeable about vaccinations and VPD. In multivariable analysis, male paediatricians, paediatricians 55 years or older, those who had participated in training courses in the previous 5 years, those who reported that taking courses and reading the scientific literature had contributed moderately or a great deal to their vaccinology knowledge and those who had implemented vaccination promotion activities, felt more knowledgeable about vaccinations compared with other paediatricians. Contrary to what was initially observed in the univariate analysis, PCP felt less knowledgeable than hospital and community paediatricians.

**Table 4:** Factors associated with feeling knowledgeable about vaccinations, multivariable logistic regression model, Italy, 2016 (n = 903)

Variables	Crude OR	95% CI	Adjusted OR	95% CI
Sex				
Female	1	Ref	1	Ref
Male	2.23	1.63–3.07	1.62	1.10–2.38
Age (years)				
35–54	1	Ref	1	Ref
> 54	3.02	2.25–4.06	2.15	1.49–3.12
Country where medical degree was obtained				
Foreign	1	Ref	NI	
Italy	0.82	0.21–3.20		
Practice location				
Medium-sized city	1	Ref	NI	
Large city	1.39	0.93–2.09		
Small city/town	1.14	0.83–1.57		
Type of paediatric practice				
Other	1	Ref	1	Ref
Primary care	1.44	1.06–1.97	0.61	0.40–0.95
Vaccine courses or conferences in previous 5 years				
No	1	Ref	1	Ref
Yes	3.00	2.12–4.25	2.16	1.34–3.49
Vaccinology training				
No	1	Ref	NI	
Yes	1.31	0.96–1.77		
Years since specialty certification				
0–5	1	Ref	NI	
6–15	1.62	0.81–3.24		
16–23	3.10	1.63–5.89		
> 23	5.29	3.03–9.22		
Years since medical degree				
0–5	1	Ref	NI	
6–10	0.18	0.02–2.19		
11–20	0.36	0.03–4.10		
>20	1.25	0.11–1.38		
Years of activity as a paediatrician				
< 1	1	Ref	NI	
1–5	1.61	0.46–5.64		
6–10	2.31	0.65–8.24		
> 10	7.23	2.30–22.67		
Degree of influence of formal university training				
Low	1	Ref	NI	
High	0.81	0.59–1.13		
Degree of influence of the scientific literature				
Low	1	Ref	1	Ref
High	2.11	1.12–4.01	1.97	0.96–4.04
Degree of influence of conference participation				
Low	1	Ref	1	Ref
High	3.82	1.74–8.40	3.07	1.21–7.79
Degree of influence of institutional websites				
Low	1	Ref	NI	
High	1.04	0.70–1.56		
Degree of influence of non-institutional websites				
Low	1	Ref	NI	
High	1.33	0.87–2.04		
Degree of influence of discussions with other colleagues				
Low	1	Ref	NI	
High	1.58	1.03–2.41		
Administers vaccines				
No	1	Ref	NI	
Occasionally	3.60	2.10–6.17		
Regularly	1.57	1.14–2.14		
Implemented vaccination initiatives				
No	1	Ref	1	Ref
Yes	2.34	1.72–3.18	2.27	1.54–3.33

CI: confidence interval; NI: not included in the final model; OR: odds ratio; Ref: reference value.

## Discussion 

A large number of paediatricians participated in our study and their demographic and professional characteristics (mean age, male to female ratio, years in practice) closely matched those reported in the latest Statistical report of the Italian National Health Service which describes the characteristics of healthcare providers practicing in Italy by specialty [16]. In addition, the number of participating paediatricians per 100,000 paediatric population was evenly distributed throughout Italy’s 21 Regions. The study sample was therefore representative of the paediatric specialist population in Italy. The PCP who participated in the study represented 8.2% of all PCP in Italy (n = 7,705).

The most important finding of this study is that the vast majority of Italian paediatricians are favourable to vaccination and believe that children should receive all vaccines in the childhood immunisation schedule. This is in agreement with the findings of another European study that examined in 2015 the attitudes of Swiss physicians and pharmacists (including 431 paediatricians) towards immunisation and found that most paediatricians were in agreement with the Swiss universal immunisation schedule [10]. As recommended by some authors, the finding that the paediatric medical community is vastly favourable to vaccinations should be communicated to parents whenever possible as this can be an important pro-vaccine message that may increase public support for vaccination [17,18]. Interestingly, more than half of the paediatricians in our study, which we conducted before the introduction of the new Italian mandatory vaccination law, also reported being favourable to schools requiring pupils to be vaccinated.

Although most paediatricians were favourable to vaccinations in general, our study identified some gaps between their overall positive attitudes towards vaccination and their knowledge, beliefs and practices. A considerable proportion (one third) of our sample did not feel sufficiently informed about vaccines and VPD and about how to address parental concerns. Some paediatricians were found to have a falsely low perception of disease risk. In addition, we found that a relevant number held false beliefs about vaccines and expressed concerns about the safety or usefulness of vaccines. About one third reported that they did not completely trust vaccine information given by health authorities and scientific societies. These results add to similar studies conducted among healthcare workers in Europe indicating that vaccine hesitancy exists not only in the general population but also, to some extent, in HCW [7,19,20]. In particular, a qualitative study in four European countries published in 2016 reported an overall positive attitude towards vaccination among HCW but also vaccine safety concerns, questions about the need for vaccines and/or mistrust especially of pharmaceutical companies [7]. Two studies examined vaccination-related behaviours and perceptions of French general practitioners (GPs) and found a moderate prevalence of vaccine hesitancy [19,20]. Some doubts about vaccine risks were found to exist also among physicians (in this case GPs) with no or slight vaccine hesitancy, most of whom are very favourable toward vaccination in general [20].

In terms of knowledge, our study identified some knowledge gaps regarding true and false contraindications to vaccinations. False contraindications are conditions or circumstances that do not preclude vaccination but are mistakenly considered to be contraindications. It is essential that paediatricians are aware of what constitutes true and false contraindications, to be able to confidently reply to parents’ questions, avoid adverse reactions following vaccinations and avoid missing opportunities to administer recommended vaccines in a timely manner. In a recent survey conducted in 2014 among French GPs, 94% of 1,582 respondents reported that they would recommend postponing hexavalent vaccination (diphtheria, tetanus, pertussis, poliomyelitis, *Haemophilus Influenzae* type b and hepatitis B) in a child with a minor febrile illness (false contraindication); the authors called for clearer and more consistent guidelines on contraindications to vaccination [21]. In our study, age of at least 55 years was an important determinant of feeling knowledgeable about vaccinations. It is expected that older physicians feel more knowledgeable than younger paediatricians since they have acquired experience during their many years in practice. Younger paediatricians must rely almost exclusively on what they have learned during medical and residency training, while formal training in vaccinology is lacking in many paediatric residency programmes in Italy. In fact, two thirds of paediatricians who had completed their residencies 5 years or less before the survey did not feel sufficiently knowledgeable about vaccinations. Major gaps in the initial training and continuous medical education of physicians regarding vaccination have also been identified in other countries in Europe [21]. Vaccinology courses, including courses in communication, should be part of the university core curriculum for all future health professionals and of compulsory continuing medical education requirements for health professionals involved in vaccinations [22].

In agreement with data from the literature, having participated in training courses in the previous 5 years was another determinant of feeling knowledgeable about vaccinations [6,9,19]. In a recent review of studies on vaccine hesitancy among healthcare providers, knowledge about vaccines and vaccine efficacy and safety was found to contribute to providers’ confidence and increase their willingness to recommend vaccination [6]. Another study conducted in December 2013 to January 2014 among 218 paediatric providers in Israel (92% nurses, 8% paediatricians) found that increasing their knowledge and addressing their concerns about vaccination improved their adherence to the routine immunisation programme regarding their own children [9]. Finally, a study conducted in 2014 among general practitioners in France showed that physicians recommended vaccines frequently when they felt comfortable explaining their benefits and risks to patients or trusted official sources of information highly [19].

It is interesting to note that in our study, ca 70% of paediatricians who did not feel knowledgeable had in fact attended training courses. These results suggest that although vaccination training courses are widespread, their contents may need to be more focused on vaccine safety, false contraindications and how to respond to parents’ concerns.

The interaction between paediatricians and parents is important in building and maintaining confidence in the vaccination programme and maintaining high levels of vaccination uptake, and paediatricians should be more proactive in initiating the conversation about vaccines with parents rather than waiting for them to raise specific questions or concerns. A recent study (2016) evaluating Italian parents’ attitudes towards vaccination, found that only 84% of parents had received a recommendation from their paediatrician to have their children vaccinated with all vaccines included in the national vaccination schedule. In the same study, not having received a recommendation was found to be a determinant of vaccine hesitancy, confirming the crucial role of PCP in influencing parental choice about vaccination [2,19,23-25]. According to more than half of the interviewed parents, information provided by HCW should highlight not only vaccination benefits but also risks.

Our study showed that about one third of paediatricians do not systematically verify their patients’ vaccination status. In Italy, PCP but not hospital paediatricians are expected to verify their patients’ vaccination status during the periodic health evaluations; however, 28% of PCP in our survey did not systematically do this. This is an important system barrier to achieving and maintaining vaccination uptake: vaccination checks should become common practice for both primary care and hospital paediatricians, and children whose vaccinations are not up to date should be referred to the local vaccination clinic.

Most Italian paediatricians reported having encountered some type of vaccine refusal in their practices and the vast majority said they would try to change the minds of parents’ who refuse vaccinations; however, they do not seem to consider it equally important to change parents’ minds about delaying vaccinations. Delaying vaccinations leaves children susceptible to preventable diseases for an unjustifiable longer period of time and should be discouraged. This issue should be highlighted in vaccination courses.

This survey was self-completed and we cannot exclude that paediatricians who are sceptical about vaccinations may have decided not to participate in the study. On the other hand, it is also possible that because the questionnaire was anonymous, they may have decided to participate in order to have a chance to express their opinions. In addition, with behaviours being self-reported, desirability biases cannot be excluded. Finally, our results are context-specific and may not be generalisable to paediatricians in other countries.

## Conclusions 

The vast majority of Italian paediatricians are favourable to vaccination. However, gaps were identified between their overall positive attitudes and their knowledge, beliefs and practices. A significant proportion, particularly of younger paediatricians, does not feel sufficiently knowledgeable about vaccine safety and how to address parent’s questions. To maintain high levels of vaccination uptake, paediatricians must be familiar with risks of VPD, vaccine safety, and false contraindications, dispel any doubts they themselves may have regarding false myths and be able to effectively communicate information about vaccines to parents. Our results will be useful when developing targeted interventions to increase paediatricians’ knowledge about vaccinations and their confidence in addressing parents’ concerns. There is also a need to strengthen paediatricians’ trust in the health authorities; this can be achieved through transparent, complete and accurate information and recommendations about vaccines and VPD and through increased involvement of paediatricians in the decision-making process regarding vaccination strategies. Finally, it is necessary to reduce system barriers to achieving and maintaining vaccination uptake, through a uniform approach or guidance on regularly checking vaccination status and evidence-based interventions such as reminder/recall systems.

## Supplementary Data

846000 Supplement
