# Supplementary material for: Childhood vaccinations: knowledge, attitudes and practices of paediatricians and factors associated with their confidence in addressing parental concerns, Italy, 2016
Source: Euro Surveill. 2019 Feb 7;24(6):1800275. doi: 10.2807/1560-7917.ES.2019.24.6.1800275 (PMC6373065; doi:10.2807/1560-7917.ES.2019.24.6.1800275)
Supplement: Supplement [file 1800275_FILIA_Supplement.pdf]

**Disclaimer.** This supplementary material is hosted by *Eurosurveillance* as supporting information alongside the article “Childhood vaccinations: knowledge, attitudes and practices of paediatricians and factors associated with their confidence in addressing parental concerns, Italy, 2016” on behalf of the authors who remain responsible for the accuracy and appropriateness of the content. The same standards for ethics, copyright, attributions and permissions as for the article apply. *Eurosurveillance* is not responsible for the maintenance of any links or email addresses provided therein.

## Survey on knowledge, attitudes and practice of Italian pediatricians in relation to vaccinations

Dear Doctor,
We kindly ask your participation in a brief survey regarding the prevention of vaccine-preventable diseases. The aim of the survey is to gather information regarding knowledge and attitudes of pediatricians towards vaccinations. The questionnaire is simple and will take about 15 minutes to complete. It is an anonymous questionnaire and we assure you that your responses will be treated with complete confidentiality; responses to this survey will be presented only in an aggregated format for the purpose of study and research. Thank you for your time and availability.

### SECTION 1

**1) In my professional activity,**

- ☐ I advise parents to vaccinate their children according to the vaccination schedule
- ☐ I recommend only mandatory vaccinations
- ☐ I do not recommend vaccinations
- ☐ I do not express an opinion regarding vaccinations (neutral attitude, neither in favor nor against vaccinations)

**2) In your professional activity, do you administer childhood vaccinations?**

- ☐ Yes, regularly
- ☐ Yes, occasionally
- ☐ No, never

**3) In your professional activity how frequently do you talk to families about the importance of vaccinations?**

- ☐ On every available occasion
- ☐ Occasionally, when the topic comes up
- ☐ Only if parents bring up the topic of vaccination
- ☐ Never, but I am in favor of vaccination
- ☐ Never, I am against vaccinations

**4) What is your reaction towards parents who refuse to vaccinate their child for one or more diseases (only one answer allowed):**

- ☐ I support their decision
- ☐ I try to change their minds by providing information on vaccines and disease risks
- ☐ I do not interfere with their decision
- ☐ I have never been faced with this situation

**5) What is your reaction towards parents who decide to delay one or more vaccinations for their child under two years of age (only one answer allowed):**

☐ I support their decision
☐ I try to change their minds by providing information on vaccines and disease risks
☐ I do not interfere with their decision
☐ I have never been faced with this situation

**6) In the last year have you carried out any vaccination promotion initiatives among your patients?**

☐ No, but I am in favor of vaccination
☐ No, I am against vaccination
☐ Yes, posters or vaccine information materials in waiting room
☐ Yes, I send reminders by phone or e-mail
☐ Yes, other (specify): ________________________________________________)

**7) During the last 2 years, have you perceived any changes in the level of parental concern regarding the safety and/or efficacy of vaccines:**

☐ Yes, it has increased a great deal
☐ Yes, It has slightly increased
☐ No, it has not changed
☐ Yes, it has slightly decreased
☐ Yes, it has decreased a great deal

**8) According to your professional experience, which of the following is the single most important reason parents decide to refuse one or more vaccines for their child? (only 1 answer is possible)**

☐ Vaccine safety concerns
☐ Concerns that vaccines can weaken your child's immune system
☐ Belief that the probability of contracting a vaccine-preventable disease is very low
☐ Belief that vaccine-preventable diseases are harmless and not serious enough to justify vaccination
☐ Belief that vaccines are not effective
☐ Belief that vaccine recommendations are guided by profit considerations of pharmaceutical companies
☐ Religious reasons
☐ I don't know
☐ Other (please specify: ________________________________________________)

**9) In your professional activity, have you ever witnessed any of the following vaccine-preventable diseases?**

| | | |
|---|---|---|
| Pertussis | ☐ Yes | ☐ No |
| Tetanus | ☐ Yes | ☐ No |
| Measles | ☐ Yes | ☐ No |
| Mumps | ☐ Yes | ☐ No |
| Rubella | ☐ Yes | ☐ No |
| Varicella | ☐ Yes | ☐ No |
| Hepatitis B (acute or chronic infection) | ☐ Yes | ☐ No |
| Invasive pneumococcal disease/meningococcal disease/ Hib | ☐ Yes | ☐ No |

**10) In your professional activity, have you ever witnessed any of the following serious adverse events that you believe were caused by vaccination?**

Seizures ☐ Yes ☐ No
Anaphylaxis or any other important allergic reaction ☐ Yes ☐ No
Neuritis/neuropathy ☐ Yes ☐ No
Multiple sclerosis ☐ Yes ☐ No
Encephalopathy/Encephalitis ☐ Yes ☐ No
Autism ☐ Yes ☐ No
Guillain Barré Syndrome ☐ Yes ☐ No
Other serious adverse events ☐ Yes ☐ No
(please specify: ____________________________________)

**11) How often do you verify that your patients are up to date with the vaccination schedule?**

☐ systematically ☐ often ☐ occasionally ☐ never

**12) Do you have an idea of what proportion of your pediatric patients are unvaccinated?**

☐ < 5% ☐ 5-10% ☐ >10% ☐ I don't know

**13) In your opinion, how likely is it that an unvaccinated child living in Italy will contract one of the following diseases in the next 10 years?**

| | **Very unlikely** | **Unlikely** | **Not very likely** | **Likely** | **Very likely** | **I don't know** |
|---|---|---|---|---|---|---|
| Diphtheria | | | | | | |
| Pertussis | | | | | | |
| Tetanus | | | | | | |
| Measels | | | | | | |
| Mumps | | | | | | |
| Rubella | | | | | | |
| Polio | | | | | | |
| Varicella | | | | | | |
| Invasive pneumococcal disease/meningococcal disease/*Haemophilus influenzae* b | | | | | | |

**14) In your opinion, if a child were to get one of these diseases, how likely is it that he/she would become seriously ill?**

| | **Very unlikely** | **Unlikely** | **Not very likely** | **Likely** | **Very likely** | **I don't know** |
|---|---|---|---|---|---|---|
| Diphteria | | | | | | |
| Pertussis | | | | | | |
| Tetanus | | | | | | |
| Measels | | | | | | |
| Mumps | | | | | | |
| Rubella | | | | | | |
| Polio | | | | | | |
| Varicella | | | | | | |
| Invasive pneumococcal disease/meningococcal disease/*Haemophilus influenzae* b | | | | | | |

**15) How effective are the following vaccines in preventing disease in children?**

| | Very effective | Effective | Not very effective | Not at all effective | I don't know |
|---|---|---|---|---|---|
| Diphteria-Tetanus-acellular Pertussis (DTPa) | | | | | |
| Haemophilus b (Hib) | | | | | |
| Measles-Mumps-Rubella (MMR) | | | | | |
| Inactivated Polio (IPV) | | | | | |
| Pneumococcal - conjugate vaccine | | | | | |
| Hepatitis B (HBV) | | | | | |
| Varicella (VZV) | | | | | |
| Meningococcal C | | | | | |
| Influenza | | | | | |
| Human Papilloma Virus (HPV) | | | | | |

**16) Your patient is scheduled to receive the second dose of hexavalent vaccine; which of the following conditions do you consider to be a contraindication?**

| | False contraindication | Temporary contraindication | Permanent contraindication | Don't know |
|---|---|---|---|---|
| Severe allergic reaction to a previous dose, including anaphylaxis | | | | |
| Fever following a previous dose | | | | |
| Acute severe gastroenteritis | | | | |
| Otitis media, without fever | | | | |
| Family history of reaction following a pertussis vaccine dose | | | | |
| Acute upper airway infection without fever | | | | |
| History of pertussis | | | | |
| Diagnosis of epilepsy, well controlled | | | | |
| Fever 38-40°C and moderate illness | | | | |
| Fever >40°C and severe illness | | | | |
| Congenital immunodeficiency | | | | |

**17) In your opinion, how safe are the following vaccines?**

| | Extremely safe | Safe | Not very safe | Not at all safe | Don't know |
|---|---|---|---|---|---|
| Hexavalent | | | | | |
| Diphtheria-Tetanus-acellular Pertussis (DTPa) | | | | | |
| *Haemophilus b* (Hib) | | | | | |

| | | | | | |
|---|---|---|---|---|---|
| Measles-Mumps-Rubella (MMR) | | | | | |
| Inactivated Polio (IPV) | | | | | |
| Pneumococcal-conjugated (PCV) | | | | | |
| Hepatitis B (HBV) | | | | | |
| Varicella (VZV) | | | | | |
| Meningococcal C | | | | | |
| Influenza | | | | | |
| HPV | | | | | |

**18) In general, you are:**

☐ absolutely against vaccinations
☐ moderately against vaccinations
☐ neutral
☐ moderately in favour of vaccinations
☐ absolutely in favour of vaccinations

**19) Please indicate if you agree with the following statements:**

| | **Fully disagree** | **Partly disagree** | **No opinion** | **Partly agree** | **Fully agree** |
|---|---|---|---|---|---|
| Vaccines are important for my patients' health | | | | | |
| If we stop vaccinating. many diseases that have become rare may re-emerge | | | | | |
| It is better for children to develop natural immunity by getting sick rather than to get a vaccine | | | | | |
| Childhood vaccines are given too early | | | | | |
| Healthy children do not need to be vaccinated | | | | | |
| I am afraid that one of my patients may develop a severe adverse reaction following vaccination | | | | | |
| Vaccines weaken or overload the immune system | | | | | |
| When children get vaccinated, the whole community benefits | | | | | |
| The second dose of MMR is useful | | | | | |
| Conditions such as autism and multiple sclerosis may be caused by vaccines | | | | | |
| Allergies are on the rise because of vaccinations | | | | | |
| I am favorable to introducing mandatory school immunisation requirements | | | | | |
| In the U.S.A. paediatricians are increasingly rejecting patients whose parents refuse vaccinations. I agree with this attitude. | | | | | |

| | | | | | |
|---|---|---|---|---|---|
| Vaccine policy is influenced by financial profits of pharmaceutical companies | | | | | |
| Children receive too many vaccines | | | | | |
| Vaccines are among the safest and most tested medicinal products | | | | | |
| The frequency of adverse events to vaccines is underestimated | | | | | |
| Vaccination is cost-effective | | | | | |
| Vaccine information provided by health authorities and scientific societies is reliable | | | | | |

**20) Do you prescribe homeopathy to your patients?** ☐ Often ☐ No ☐ Occasionally

**SECTION 2**

**21) Age (years):** ☐ < 35 ☐ 35 – 44 ☐ 45 – 54 ☐ 55 – 64 ☐ > 64

**22) Gender:** ☐ M ☐ F

**23) In what year did you receive your medical degree?** ___________

**24) Country of graduation:** ☐ Italy ☐ Other, specify:______________________

**25) In what year did you obtain pediatric specialty certification?** Year: ☐☐☐☐

**26) In what province do you practice as a pediatrician?** _______________

**27) What type of professional activity do you practice?**

☐ Primary care pediatrician

☐ Community Pediatrician

☐ Hospital-based Pediatrician

☐ Pediatrician in private practice

☐ Retired

**28) Years in practice?**

☐ < 1 ☐1- 5 ☐ 6 – 10 ☐ >10

**29) Which of the following best describes the type of area where most of your patients reside?**

☐ Large City (> 250.000 population)

☐ Medium-sized city (50.000-250.000 population )

☐ Small city/town (<50.000 population)

**30) Did you receive any training in vaccinology during your pediatric specialty training?**

☐ Yes ☐ No ☐ I don't remember

**31) Do you feel sufficiently knowledgeable about vaccines and vaccine-preventable diseases, including incidence, complications, benefits/risks of vaccination and contraindications to vaccination, to be able to confidently address parental questions?**

☐ Yes ☐ No

**32) If not, which topics would you like to receive further training in and in which order of priority?**

☐ Epidemiology and complications of vaccine-preventable diseases
☐ Vaccine efficacy
☐ Vaccine safety and risks
☐ Vaccine components
☐ Pharmacovigilance
☐ Contraindications and precautions of vaccinations
☐ How to respond to parents’ most frequent concerns about vaccinations
☐ Communication methods on vaccination issues

**33) Which other topic that was not mentioned in the previous question would you like to receive further training in?**

________________________________________________

**34) How much do/ have the following means influence/influenced your knowledge on vaccine-preventable diseases?**

| | **A great deal** | **Moderate amount** | **Neutral** | **Not much** | **Not at all** |
|---|---|---|---|---|---|
| University training | | | | | |
| Scientific literature | | | | | |
| Conferences, meetings and training courses | | | | | |
| Institutional vaccine web pages | | | | | |
| Non-institutional vaccine web pages | | | | | |
| Discussions and contacts with colleagues | | | | | |

**35) In the past 5 years, have you attended vaccination training courses or conferences (including distance learning and online courses)?**

☐ Yes If yes, specify the number of courses/conferences: ☐ 1-3 ☐ 4-6 ☐ 7-9 ☐ > 10

☐ No

**36) Through which of the following scientific associations have you been invited to participate in the present survey? (more than one answer is possible)**

☐ Cultural Association of Pediatricians (ACP)
☐ Italian Federation of Pediatricians (FIMP)
☐ Italian Association of Pediatricians (SIMPe)
☐ Italian Pediatric Association (SIP)
☐ Italian Association of Social Pediatrics (SIPS)

***Thank you for your participation in this survey.***

*If you have any comments, please add them here:*

________________________________________________
________________________________________________
________________________________________________
________________________________________________
________________________________________________
________________________________________________
